# Plasmonic Approaches for the Detection of SARS-CoV-2 Viral Particles

**DOI:** 10.3390/bios12070548

**Published:** 2022-07-21

**Authors:** Sabine Szunerits, Hiba Saada, Quentin Pagneux, Rabah Boukherroub

**Affiliations:** University of Lille, CNRS, Centrale Lille, University Polytechnique Hauts-de-France, UMR 8520-IEMN, F-59000 Lille, France; hiba.saada@univ-lille.fr (H.S.); quentin.pagneux@univ-lille.fr (Q.P.); rabah.boukherroub@univ-lille.fr (R.B.)

**Keywords:** SARSC-CoV-2, diagnostics, surface plasmonic resonance (SPR), spike protein, point-of-care testing

## Abstract

The ongoing highly contagious Coronavirus disease 2019 (COVID-19) pandemic, caused by severe acute respiratory syndrome coronavirus 2 (SARS-CoV-2), underlines the fundamental position of diagnostic testing in outbreak control by allowing a distinction of the infected from the non-infected people. Diagnosis of COVID-19 remains largely based on reverse transcription PCR (RT-PCR), identifying the genetic material of the virus. Molecular testing approaches have been largely proposed in addition to infectivity testing of patients via sensing the presence of viral particles of SARS-CoV-2 specific structural proteins, such as the spike glycoproteins (S1, S2) and the nucleocapsid (N) protein. While the S1 protein remains the main target for neutralizing antibody treatment upon infection and the focus of vaccine and therapeutic design, it has also become a major target for the development of point-of care testing (POCT) devices. This review will focus on the possibility of surface plasmon resonance (SPR)-based sensing platforms to convert the receptor-binding event of SARS-CoV-2 viral particles into measurable signals. The state-of-the-art SPR-based SARS-CoV-2 sensing devices will be provided, and highlights about the applicability of plasmonic sensors as POCT for virus particle as well as viral protein sensing will be discussed.

## 1. Introduction

Infection with the recent coronavirus COVID-19 leads to severe illness, which derives from the host’s immune response, especially the release of a storm of pro-inflammatory cytokines. This cytokine storm produces extreme inflammatory and immune responses, especially in the lungs, leading to acute respiratory distress. Hope that SARS-CoV-2, the virus that causes COVID-19, becomes endemic over time is still pending. Widespread vaccination has contributed to fewer people becoming infected and hospitalized, ultimately alleviating the burden of COVID-19. Vaccines play a critical role in preventing deaths and hospitalization caused by this infectious disease and are contributing to controlling the spread of the disease. However, both vaccinated and nonvaccinated people need to remain aware of the additional protective behaviors required to control the pandemic. Several strategies were implemented to combat COVID-19, including wearing masks, hand hygiene and social distancing [1]. The impact of these strategies on COVID-19 remains largely unclear. However, a recent meta-analysis demonstrated that face mask use was associated with an 85% reduced risk of developing clinical symptoms of the viral infection causing COVID-19 [2].

Next to vaccination and protection strategies, the implementation of an early diagnostics of people infected with COVID-19 has proven to be crucial to the COVID-19 pandemic management. There are mainly three major methods for the detection of SARS-CoV-2 infection [3]. Molecular tests, such as polymerase chain reaction (PCR) approaches, are highly sensitive and specific for detecting viral RNA and are recommended for those symptomatic and for activating public health measures. Lateral-flow-based antigen rapid detection assays [4] detect viral proteins and, although less sensitive than the molecular tests, have the advantages of being cheap, fast and easy to be performed by any individual. Antigen rapid detection tests, mainly in the form of lateral flow devices, can be used as a public health tool for screening individuals at enhanced risk of infection, to protect people who are clinically vulnerable, to ensure safe travel and the resumption of schooling and social activities, and to enable economic recovery [3]. Realistically, the expansion of regular testing relies on the development of fast, low-infrastructure testing or self-testing, such as antigenic rapid tests with a sensitivity comparable to that of PCR [5]. Such COVID-19 diagnostic tests will continue to play a crucial role in the transition from pandemic response to pandemic control.

**Figure 1:**
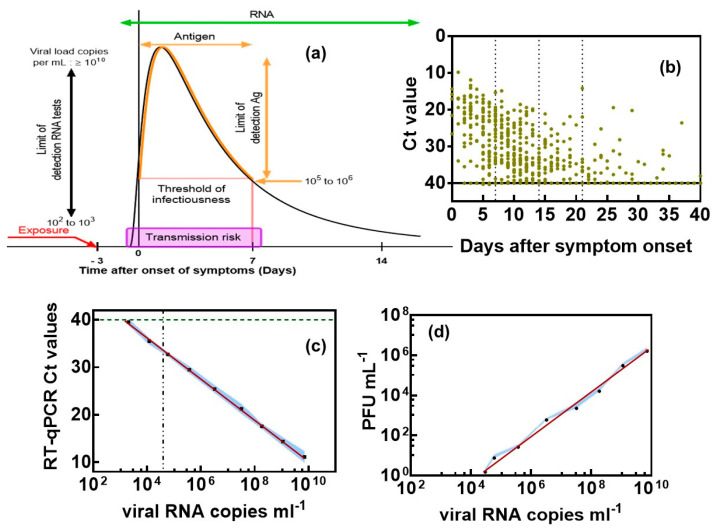
Clinical significance of Ct values and correlation with viral RNA copies as well as plaque-forming units (PFU): (**a**) Timeline of SARS-CoV-2 infectivity taking into account our own findings and those of others [12,14]. (**b**) Ct values as a function of time after symptom onset in nasopharyngeal swab specimens of COVID-19 patients. (**c**) Correlation of Ct counts with viral RNA copies. (**d**) Correlation of viral RNA copies mL^−1^ with plaque-forming units (PFU) of SARS-CoV-2 as a measure of infectivity. Vero E6 cells were infected with 10-fold dilutions of a SARS-CoV-2 isolate clade 20A.EU2 (EU variant). Calculation of estimated virus concentration was carried out by the Spearman and Karber method and expressed as TCID50/mL (1 pfu mL^−1^ = TCID50/mL × 0.7). The results are expressed as the mean ± SEM of at least three independent measurements for each group.

Concerns about the reduced sensitivity of lateral flow antigenic tests in comparison to PCR have resulted in the consideration of alternative approaches and concepts [6,7]. To evaluate the quality of these new diagnostic concepts, it is primordial to define a target sensitivity in terms of the minimal viral particles per mL concentration to be sensed, how this value correlates to plaque-forming units per mL (PFU mL^−1^) and what the correction to cycle threshold (C_t_) values from RT-PCR could be. It is believed that infectiousness begins 2–3 days prior to symptoms onset, with people being most infectious around the time of symptom onset (Figure 1a) [8]. Asymptomatic and symptomatic SARS-CoV-2 infections can have different characteristic time scales of transmission, with a mean infectious period of about 9–10 days for asymptomatic individuals [9,10,11] compared to symptomatic ones of about 1–4 days [12].

One fundamental issue in considering viral diagnostics sensitivity is consequently related to the question of how to compare/relate cycle threshold (Ct) values form RT-PCR obtained from different protocols and viral samples [13]. This exercise remains complex, as Ct values can only be interpreted correctly by having an idea about the health history of the patient [14]. The uncertainty about the range of viral loads that constitute a transmission risk is an additional factor when considering Ct cut-off values and diagnostic sensitivity [15]. People are most infectious around the time of symptom onset (Figure 1a), for whom the viral load in the upper respiratory tract is the highest [8]. Asymptotic individuals follow a similar dynamic and contribute in the same manner as pre-symptomatic individuals to the viral spread. There is a general agreement that Ct values are linked to SARS-CoV-2 viral load, with Ct of 33–35 being associated with low infectivity, Ct value < 20 being linked to high viral load and Ct = 40 being the cut-off between positively and negatively identified individuals. The timeline of SARS-CoV-2 RNA was lately confirmed by some of us [16] using data from 520 COVID-19 patients (Figure 1b). The lowest Ct values, corresponding to the highest virus loads, were recorded early after symptom onset, followed by a decline in virus load with increasing time after symptom onset.

To correlate Ct values with the absolute number of virions, the number of viral RNA copies can be determined in parallel (Figure 1c). As expected, a linear relation between RT-PCR Ct values and viral RNA copies mL^−1^ was observed. A Ct value thus corresponds to 2.1 × 10^3^ viral RNA mL^−1^, while a Ct = 12 correlates with 7.1 × 10^9^ viral RNA mL^−1^. The presence of viral RNA does not necessarily imply the presence of infectious virions. Virions could be defective (e.g., by mutation) or might have been deactivated by environmental conditions. Therefore, the use of viral RNA copies as an approximation for the number of infectious viral particles leads to an overestimation. It is important to keep this caveat in mind when interpreting the data about viral loads. Nevertheless, for many viruses, even a small dose of virions can lead to infection. For the common cold, for example, ~0.1 TCID_50_ is sufficient to infect half of the exposed people [17]. To assess the concentration of infectious viruses, the 50% tissue-culture infectious dose with 1 PFU mL^−1^ = TCID_50_/mL × 0.7 has to be determined by infecting replicate cultures of susceptible cells with dilutions of the virus and noting the dilution at which half the replicate dishes become infected. Figure 1d indicates that 2.1 × 10^3^ viral particles mL^−1^ results in no palatable virus. The onset for forming 1 PFU mL^−1^ corresponds with a minimal viral particle load of (4.0 ± 1.9) × 10^4^ viral particles mL^−1^. This correlates with about Ct = 32 ± 1. In a recent wok by Pickering et al. [6], Ct values of 30 were correlated to 1 PFU mL^−1^ and 5 × 10^4^ RNA viral particles mL^−1^. The viral particle load correlates extremely well with our findings. The difference in Ct values is linked to the different fragments being used, i.e., the N gene by Pickering et al. [6] and IP targets by us [18]. Such benchmarking is of high importance for evaluating novel sensing approaches and their performance level. For RT-PCR, 100 copies of viral RNA per mL corresponds with a positive result. In addition, serological tests can provide valuable information on the immune response and are a good complement to SARS-CoV-2 RNA test. In fact, as a patient recovers, the viral load starts to decrease, and immunoglobulin levels increase until about 10 days after symptom onset. Serological tests can be performed at this timepoint.

**Figure 2:**
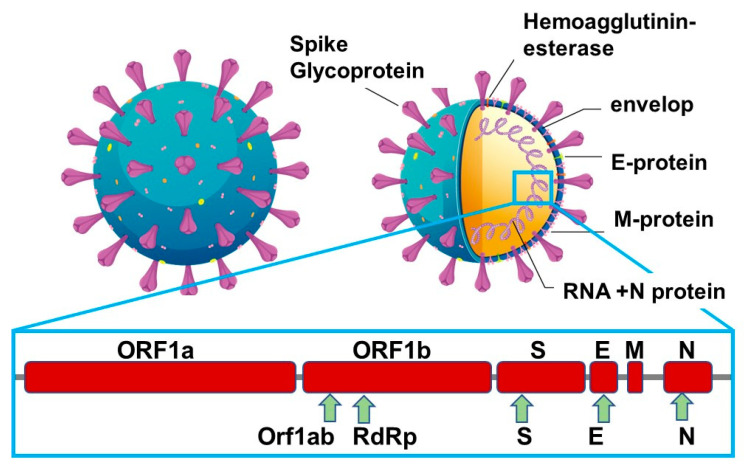
Structural proteins of SARS-CoV-2 for sensing: Viral replication requires other auxiliary genes, including open reading frame 1a (ORF1a), ORF1b and RNA-dependent RNA polymerase (RdRp).

SARS-CoV-2 causes mild or asymptomatic disease in most cases; however, severe to critical illness occurs in a small proportion of infected individuals, with the highest rate seen in people older than 70 years. Compared to other viruses, SARS-CoV-2 has a medium reproduction rate of *R*_0_ = 2.5 compared with *R*_0_ = 2 0–3 0 for SARS-CoV and the 1918 influenza pandemic, *R*_0_ = 0·9 for MERS-CoV and *R*_0_ = 1·5 for the 2009 influenza pandemic [19]. It is generally true that for a rapid transmitted disease, such as SARS-CoV-2, the most efficient way to curb its spread is early detection to isolate patients. The gold standard for COVID-19 diagnosis is nucleotide-based testing (qRT-PCR) of viral RNA in nasopharyngeal swabs, collected from the upper respiratory tracts of suspected individuals. Next to viral ssRNA, most FDA-approved commercial antigen kits target the nucleocapsid (Figure 2).

The structural proteins of SARS-CoV-2 are next to the spike glycoproteins (S1, S2), the envelop (E), the membrane (M) and the nucleocapsid (N) proteins. The M protein is the most abundant protein on the viral particles, with the E protein being the smallest major structural protein of viral particles. The S envelop protein consists of two functional subunits, S1 and S2; the S1 subunit binds to the host cell receptors, while the S2 subunit fuses with the viral and cell membranes. The S-protein remains the main target for neutralizing antibody treatment upon infection and the focus of vaccine and therapeutic design. It is also a major target for the development of diagnostic approaches but has not been widely integrated into commercial antigen kits, which are mainly based on targeting the nucleocapsid protein. The N-protein is indeed the main structural protein and responsible for the replication and transcription of the viral RNA, the packaging of the enveloped genome into viral particles and interaction with the cell cycle of host cells. It is also the most abundant protein produced and released during viral infections and can be detected in serum and urine within the first hours of infection, reaching a maximum at about 10 days after infection. In addition, only about 100 spike trimers are present on each SARS-CoV-2 virion, with an estimated total of 300 monomers, which can be targeted for sensing, while around 1000 copies of the nucleocapsid are expressed in each virion [17,20]. A comparison was recently implemented using monoclonal anti-spike antibodies [21] in an in-house-developed antigenic test for SARS-CoV-2 and a comparable test targeting the nucleocapsid protein [20] using, in particular, a novel monoclonal antibody with an affinity constant K_D_ = 0.7 nM. The antigen choice in most commercial assays, the nucleocapsid was confirmed with higher sensitivity than the spike-based assay. The spike-based assays were, however, significantly more specific than the nucleocapsid-based ones. As escape mutants have found to be manifested in these spikes as well as in the nucleocapsid proteins, a combination of both antigens on the same diagnostic device might be the way to go forward and strengthen the reliability of COVID-19 tests, an approach recently proposed by Cai et al. [22]. So, where are we standing in terms of alternatives to enzyme-linked immunosorbent assay (ELISA) and PCR using S- and N-protein targets?

This review can be seen as an addition to other ones [23,24], with recent results on clinical samples [25], underlining the high potential of portable SPR as a viral diagnostic device. A special focus will be on the potential of SPR to characterize affinity constants between bioreceptors and COVID-19 targets, an aspect often not described in more detail. However, localized surface plasmon resonance (LSPR) sensors will not be discussed, and voluble information can be found in the paper by Takemura [23]. The review will focus mainly on the detection of SARS-CoV-2 viral particles by SPR. While genes remain one of the most widely used viral biomarkers, and more sensitive and novel methods for the detection of viral genes have been implemented [26,27,28,29], such as CRISPR-associated protein 9 combined with SPR [28], we believed that molecular testing focusing on the presence of SARS-CoV-2 proteins, such as S- and N-proteins, to identify those individuals who are infected at the time of testing is more effective in directly correlating with infectivity if performed in a quantitative or at least semi-quantitative manner. In the discussion, which follows, viral-particles-based SPR sensing will be focused upon.

## 2. Surface Plasmon Resonance as a Tool for Binding Kinetics Analysis

The key to biological ligand development is understanding the binding interaction strength between the bioreceptor and the target (analyte) of interest. Classical biochemical approaches, such as Western blots, and co-immunoprecipitation approaches, only tell whether binding is occurring among biomolecules. ELISA provides more detailed information, such as binding affinity, but not without complicated and time-consuming enzyme-based amplification and labeling steps. The advantage of SPR, commercially available for more than 30 years [30], is that it uncovers accurately binding interactions in a label-free manner. In the classical gold-prism-based SPR approach, this information is obtained by flowing the analyte over the SPR prism modified with bioreceptors. The accumulation of analytes onto the sensor’s surface due to bioreceptor–analyte interactions results in an increase in the refractive index near to the sensor surface, leading to changes in SPR conditions in real time and providing information about the binding efficiency in minutes. The approach requires minimal amounts of sample for binding kinetics experiment and provides information on the rates of association and dissociation events without the use of fluorescent, magnetic or radioactive labels. A handful of different bioreceptors can be integrated on SPR sensors using different surface chemistry approaches [31], ranging from the use of classical antibodies and engineered antibodies [25] to DNA [32], aptamers [33], sugars [34], etc. The cost and complexity of SPR analysis have been largely decreased in recent years with the advent of access to affordable and portable SPR technologies [25,35,36]. SPR methods remained, however, up to recent achievements, useless for the detection of single viral particles and low viral particle concentration in general. As their prompt detection and quantification remain extremely important for precise disease diagnostics, as exemplified for COVID-19, different efforts in this direction have been described recently and will be discussed in more detail in the following.

**Figure 3:**
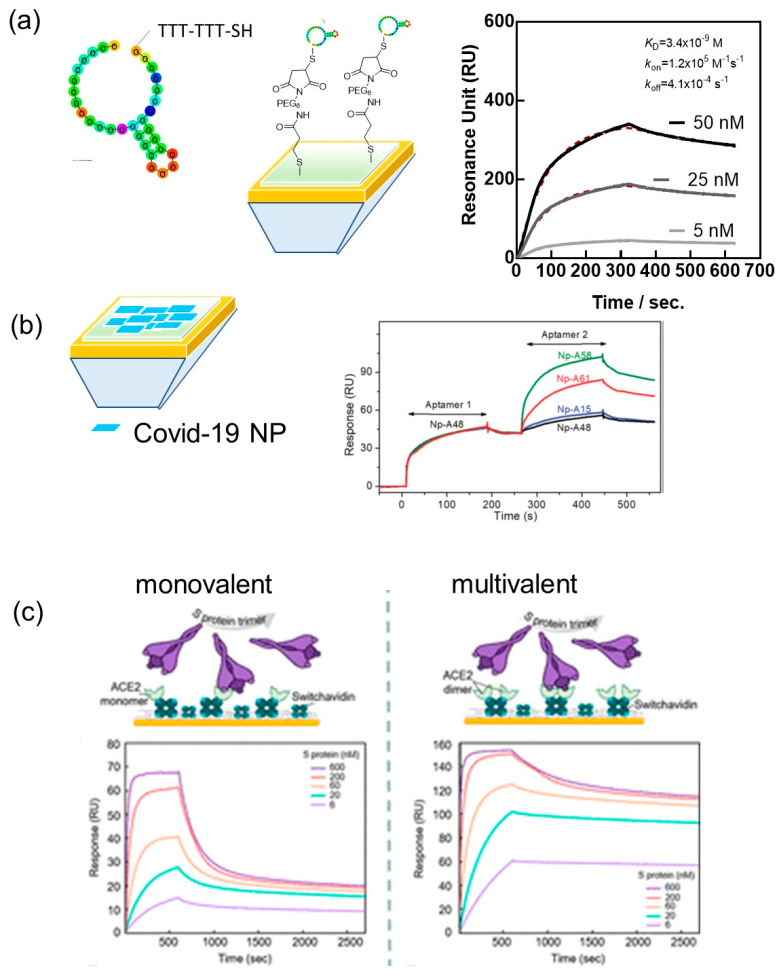
SPR as a valuable tool for the determination of affinity between SARS-CoV-2 bioreceptors and viral proteins: (**a**) SPR sensogram of the binding kinetics for S1 spike protein to 20-base aptamer “CFA0688T” from BasePairBio together with surface chemistry architecture. (**b**) SPR sensograms of the binding kinetics of N-protein specific aptamers to SARS-CoV-2 N-protein modified SPR chips (CM5 chip using EDC/NHS chemistry) with a sequence of different aptamers flown over the sensor chip (Reprinted with permission from Ref. [41]), 2020, RSC, (**c**) Schematic of SPR assay on monolayer and dimer ACE-2 modified SPR chips together with binding kinetics (Reprinted with permission from Ref. [42], 2021, ACS).

SARS-CoV-2 viral particles have a reported isoelectric point pI of 10.07 and are positively charged at physiological pH [37]. Non-specific interaction with the negatively charged backbones of aptamers might occur, requiring the design of highly specific bioreceptors. A handful of SARS-CoV-2 aptamers targeting the spike protein [38,39,40] as well as the N-protein [41] have indeed been reported. In this case, and others, SPR proved to be an efficient tool for understanding the affinity between the receptor binding domains (RBD) and the full S1 protein of SARS-CoV-2 and the surface bioreceptor, preferentially immobilized on the surface of the SPR chip to make the binding kinetics analysis comparable to future plasmonic sensing. In the case of the 20-base aptamer “CFA0688T” (Base Pair Bio) with one loop modified on the 5′ end with a thiol-TTT-TTT to give the aptamer some flexibility for its anchoring onto gold interfaces, the binding affinity to the recombinant SARS-CoV-2 S1 spike protein was determined as *K*_D_ = 3.4 ± 0.2 nM (R^2^ = 0.9985) (Figure 3a). The attachment of the SARS-CoV-2 aptamer to gold SPR chips was based on maleimide-thiol chemistry by first coupling 3-mercaptopropionic acid to the gold chip followed by EDC/NHS linking of maleimide-PEG_6_-amine (Figure 3a).

Zhang et al., reported a 58-base N-protein specific aptamer (A48) with a *K*_D_ of 0.49 nM, a *k*_on_ = 8.80 × 10^5^ M^−1^ s^−1^ and *k*_off_ = 3.48 × 10^−4^ s^−1^, as determined by SPR. However, in this experiment, the N-protein was attached to the surface using a typical EDC/NHS protocol and the aptamers flown over the surface (Figure 3b). By adopting this approach, the possibility of sandwich-type binding between different aptamers and the N-protein can be evaluated. In the first run, aptamer A48 was flown over the channel resulting in a shift of 47 RU. In the following run, a second aptamer specific to the N-protein was flown over the same channel. If this aptamer binds to different epitopes of the protein, the response signal should feature a second plateau, which was observed for A58, A61 but not for A15 and A48 as controls.

Similarly, SPR was used for the deconvolution of the avidity-induced affinity enhancement for SARS-CoV-2 spike protein and the human receptor angiotensin-converting enzyme 2 (ACE-2) [42]. Indeed, similar to other coronaviruses, the glycosylated spike proteins of the SARS-CoV-2 envelop bind to host ACE-2 receptors to mediate the fusion of the viral particles and host cell membrane. It has been shown that the chimeric structure of the SARS-CoV-2 RBD possesses higher binding affinity toward the ACE-2 compared to SARS-CoV [43]. Geschinder and co-workers [42] pointed out that the commonly considered 1:1 binding interaction between an isolated RBD of the spike protein and a single ACE-2 monomer is oversimplified and does not account for avidity effects. By designing a sensor surface favoring monovalent interaction events between the full-length S-protein and ACE-2 as well as a surface that favors the generation of multivalent effects, a *K_D_* of 60 nM was determined in the first case, while in the multivalent case, the signal accounts for a 125 nM affinity interaction (62%) but also a 4 nM affinity (28%). In the following, monomeric and multimeric ACE-2 species were linked to switch-avidin modified SPR chips, allowing resolving multiple binding events on each surface. On the dimeric ACE-2 surface, a high affinity of 283 pM was observed, mainly due to the lower *k*_off_ rate (Figure 3c).

**Figure 4:**
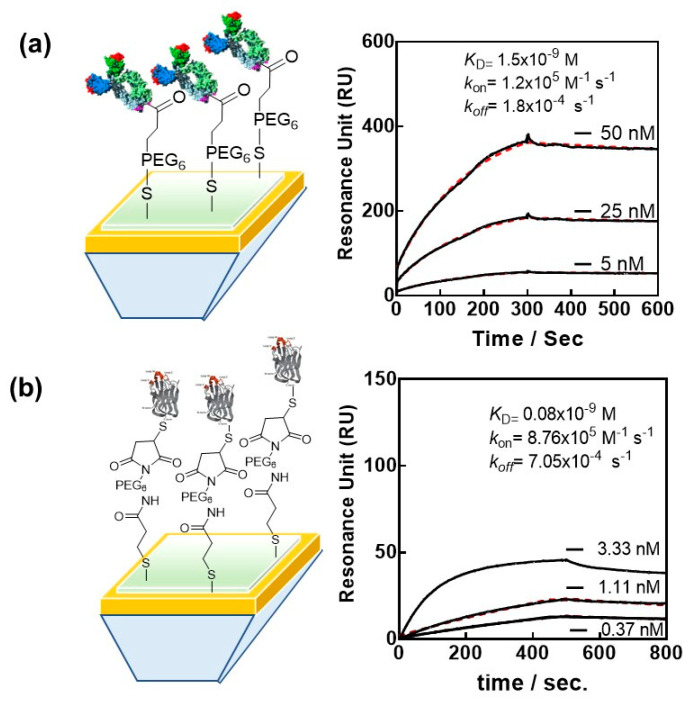
Affinity of different engineered SARS-CoV-2 antibodies: (**a**) Oriented linkage of nanobody VHH-72-Fc together with sensogram (Reprinted with permission from Ref. [25], 2022, RSC). (**b**) Binding affinities of nanoCLAMP P2712L (6His-P2710-linker-P2609-linker-Cys) to Wuhan-RBD. Gold chips were modified with a maleimide linker. Running buffer: 20 mM MOPS, 150 mM NaCl, 1 mM CaCl_2_ and 1% BSA as blocking agent (pH 6.5). Black lines depict binding data, and red lines display the 1:1 binding model fit.

Next to aptamers and ACE-2, the most widely investigated bioreceptors for SARS-CoV-2 remain the antibodies and engineered antibodies. Nanobodies have, in this respect, found a wider interest, and SPR was largely used to obtain their affinity characteristics to RBD and full-length S1 protein of SARS-CoV-2. We selected VHH-72 (PDB ID 6WAQ) [44], an anti SARS-CoV-1 anti-spike nanobody, which cross-neutralizes SARS-CoV-2, for SPR-based investigations and sensing. Despite the nanomolar affinity of VHH-72 for the SARS-CoV-2 RBD [44], the rapid dissociation is believed to negatively affect the SPR-based sensing. In addition, a common drawback of biosensors relates to the immobilization of proteins such as VHH-72 onto the transducer using EDC/NHS. Random attachment of VHH-72 is most likely to decrease the binding efficiency of a bulky target, such as the SARS-CoV-2 viral particle. Immunoglobulin or Fab fragments are the favorite binder candidates to surfaces, allowing the orientation of the nanobody’s recognition epitope toward the solution and thus the viral target. The bivalence of VHH-72-Fc, due to the Fc domain of human IgG1 genetically linked by a HHHHHHRENLYFQG linker to the VHH domain, results in nanomolar affinity constant *K*_D_ = 1.5 × 10^−9^ M with a *k*_on_ of 1.2 × 10^5^ M^−1^ s^−1^ and an improved *k*_off_ equal to 1.8 × 10^−4^ s^−1^ (Figure 4a).

More recently, novel SARS-CoV-2 RBD-specific antibody mimetics called nanoCLAMPs (nano-CLostridial Antibody Mimetic Proteins) have been investigated with SPR [45]. nanoCLAMPs, derived from an immunoglobulin-like carbohydrate binding module from a Clostridium hyaluronidase, are 4 nm × 2.5 nm antibody mimetics with distinctive advantages over other antibody mimetics as well as nanobodies. They can be screened from a naïve phage display library for high specificity target affinity in as little as 6 weeks. Their production from the cytosol of *E. coli* is cheap, with yields over 200 g/L. The high melting point >75 °C makes them stable at room temperature and thus ideal for sensor development, as the modified interfaces might be stored at room temperature over an extended period of time without any degradation of their sensing performance. The absence of other cysteine units in nanoCLAMPs makes cysteine-based surface attachment particularly easy, as reducing agents, such DTT, do not alter the protein binding structure. An affinity maturation nanoCLAMP with cysteine end, nanoCLAMP P2712 (6His-P2710-linker-P2609-linker-Cys), was lately tested and showed a *K*_D_ of 80 pM for the Wuhan RBD (Figure 4b). The ligand was covalently conjugated to gold chips modified with maleimide units via its single C-terminal Cys and, in addition, could be easily refolded on the surface following chemical denaturation with 6 M GuHCl/0.1 N NaOH.

## 3. Plasmonic Sensors of SARS-CoV-2

**Figure 5:**
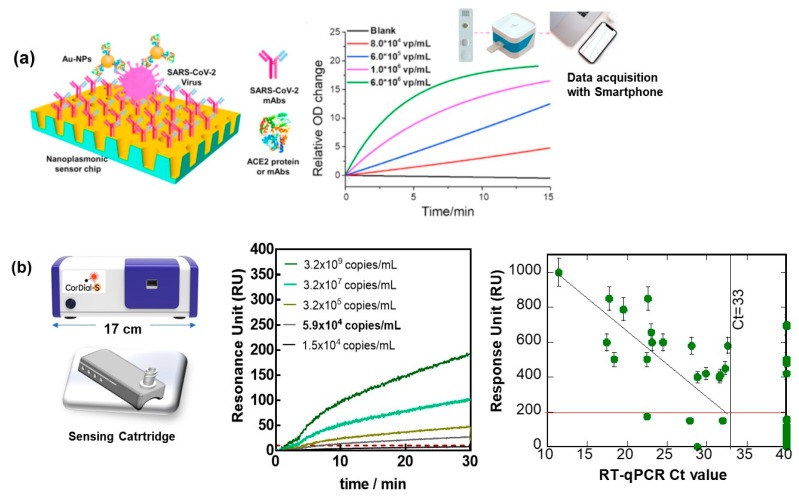
Portable SPR concepts applied to SARS-CoV-2 sensing: (**a**) Principle of nanoplasmonic resonance sensor for the detection of SARS-CoV-2 viral particles in a sandwich assay together with a photo of the developed sensor chip cartridge to be inserted into a handheld device with smartphone for data read out and binding curve to different SARS-CoV-2 pseudoviral particles concentrations (Reprinted with permission of Ref. [50], 2021, Elsevier).(**b**) (left) Image of a desk-top SPR POC testing device with cartridge-based sensing ability. (middle) SPR sensograms upon flowing cultured SARS-CoV-2 viral particles over cartridge-based SPR chip modified with VHH-72-Fc (Figure 4b), running buffer HBS-P + 1× containing 0.01 M HEPES, 0.15 M NaCl and 0.05% *v*/*v* Surfactant P20 as well as a correlation between RT-qPCR positive (50) and negative (69) nasopharyngeal samples and SPR data. Cut-off between positive and negative was 186 RIU (red line).

The development of COVID-19-specific and high-affinity biomarkers is not only useful for the design of therapeutics but has become an essential part of plasmonic SARS-CoV-2 sensors [23,46,47,48]. One of the first examples of SPR, notably intensity-modulated SPR-based virus sensing, is that reported by Chang et al. [49]. An antibody-based H7N9 virus sensing was proposed with a detection limit of 144 copies mL^−1^, a 20-fold increase in sensitivity compared with a homemade target-capture ELISA using the identical antibody. These conventional SPR testing machines were rather bulky and not adapted for implementation in clinical settings. Therefore, the SPR virus detection schemes performed in research laboratories were rarely considered as viable methods and accessible to clinical and point-of care applications. A low-cost nanoplasmonic sensor, allowing for one-step rapid detection and quantification of SARS-CoV-2 pseudoviral, was proposed by Huang et al. [50]. The concept was based on a gold nanocup array modified with antibodies; the attachment of SARS-CoV-2 to it results in a change in the plasmon resonance wavelength and intensity. Further interaction with gold nanoparticles modified with the ACE-2 protein resulted in a sensitive sandwich assay with sensing capability in the range of 10^2^–10^7^ viral particles mL^−1^ and a detection limit of 370 pseudoviral particles mL^−1^ (Table 1) within 15 min (Figure 5a). Graphene-coated SPR was proposed by Akib et al. for COVID sensing [51] with the main focus on the demonstration of the advantage of graphene SPR rather than on real sensing of virus samples.

As stated in a recent review by Jean-Francois Masson, plasmonic sensors are ideal for small and portable diagnostic devices [52]. The field has progressed lately from the use of prism-based approaches to the use of plasmonic nanomaterials, optical fibers and smartphones as optical components in the diagnostics system [53,54,55,56]. Indeed, plasmonic devices can be downscaled with limited loss in performance, as the optical measurements rely rather on wavelength or plasmonic resonance angle shift than on intensity. Signal to noise ratios remain consequently unchanged as long as the detector sensitivity is not compromised. The use of inexpensive light-emitting diode (LED) sources rather than lasers together with small USB spectrometers [57] or even smartphones [58] for read out makes the instrumentation portable and of low cost. The sensor chip can, in addition, be downscaled with no loss in analytical sensitivity, as the propagation length of plasmons is in the tens of micrometers range. The use of refractive index matching fluids, which are untidy and can interfere with the optical read out, can be avoided when disposable gold-coated prims are employed [35]. It is around sample handling where the costs of SPR and its complexity remain to be improved. The fluid handling in a portable device should be under low pressure or even without pumps required, such as passive transport of the analyte to the sensing chip [59]. Reproducible and bioreceptor-oriented surface chemistries remain, in addition, an ultimate step to be optimized for each analyte, even for portable SPR devices. The integration of deep- and machine-learning approaches to improve the detection characteristics of SPR is becoming an important and integral part for faster and sustainable sensing [25,60,61,62]. Some portable plasmonic devices had been reported, such as the smart-phone-based SPRI by Guner at al. [56], displaying refractive index changes as low as 4.12 × 10^−5^ RIU, comparable to the performance of commercial instruments as well as miniaturized platforms by PhotonicSys SPR H5 [36], Affinité Instrument [63,64] or the phase-sensitive compact IPOS-Lab SPR by Phaselab Instruments [65]. In the case of Affinité Instrument, the minimum in the spectral SPR signal is followed using a proprietary algorithm that provides a final instrumental resolution of 0.004 nm with a noise level < 5 RIU.

The use of portable SPR for diagnostics was also the focus point for studies during the COVID-19 pandemic. How to break the defect of conventional and portable SPR for their implementation in clinical settings was recently exemplified by us, using the sensing of the presence of the S1 protein of SARS-CoV-2 as an example [25]. To demonstrate how a portable SPR technology can be implemented for the sensing of SARS-CoV-2 viral particles via the S1 spike protein, we lately focused on three scientific and technological elements important for bringing SPR to the POC testing level: the oriented attachment of an engineered antibody of high affinity for the envelop S1 protein of SARS-CoV-2 and the use of a sensing cartridge, one of the first instrument considerations for achieving state-of-the-art point-of-care sensing (Figure 4b). The implementation of machine learning for predicting the cut-off value between positive and negative nasopharyngeal swab samples proved to also be essential for improving the performance of the sensor. When exposed to cultured SARS-CoV-2 viral particles (clade 20A.EU2, EU variant) of different concentrations, a sample of 5.9 × 10^4^ viral particles mL^−1^ could still be distinguished from the noise, being RU = 10 (Figure 5b), and correlated with an RT-qPCR value of around Ct = 32. To push the analysis further, the number of viral particles required to kill 50% of Vero E6 cells allowed the determination of the infectious titer and was found to be 10 PFU mL^−1^ for 5.9 × 10^4^ viral particles mL^−1.^

The clinical performance of the cartridge-based sensor was, in addition, evaluated on 50 nasopharyngeal swab samples (25 positive and 25 negative samples, as identified by RT-qPCR collected from patients at a clinical testing facility). Using a cut-off value of 186 RU (Figure 5b), from the 50 nasal swab samples that had been confirmed by RT-qPCR to be positive, 4 were identified as COVID-19 positive. With 21 samples correctly identified out of 25, in accordance with RT-qPCR, an 84% positive percentage agreement (PPA) was determined. Out of 25 nasal samples confirmed by RT-qPCR as negative, 6 were identified as negative by SPR, revealing a 76% negative percentage agreement (NPA). Using a machine-learning algorithm with 250 ms sampling time and 1 min acquisition time instead of 15 min, it was still possible to match the same results. Interestingly, the results of the cartridge-based sensor are comparable to those of SPR using microfluid channels [25]. Such work opens up the possibility of point-of-care detection of SARS-CoV-2 infection due to the unique sensitivity and lateral flow assay-comparable response time and could add strongly to virus diagnosis scenarios.

How the performance of this and other COVID-19 SPR sensors compares to other alternative portable sensing approaches can be seen from Table 1. Indeed, RT-PCR remains the most sensitive approach for viral diagnostics. Comparing an optical [25] and electrochemical sensor [18] using the same surface ligand resulted in comparable sensitivities. Both of them outperformed the lateral-flow-based assays.

**Table 1:** Comparison of different SARS-CoV-2 detection principles.

Method	Ligand Target	LoD Viral Particles mL^−1^	Ref
RT-PCR	Nucleic acid against ORF/N	<10	[66]
RT-LAMP	Nucleic acid against N	50	[67]
GFET	antibody against S1	242	[68]
Nanoplasmonic	Antibody against S1/Au-NP with ACE2	370	[50]
paper-based EC sensor	Nucleic acid	6.9 × 10^3^	[69]
Portable EC sensor	Nanobody against S1	1.2 × 10^4^	[18]
SPR	Nanobody against S1	5.9 × 10^4^	[25]
Lateral flow assays	N gene	3.0 × 10^6^	[6]

EC = electrochemical; GFET = graphene-based field effect transistor; RT-LAMP: Reverse transcription loop-mediated isothermal amplification.

## 4. Conclusions and Perspectives

Currently, various commercial POCT devices have been developed for the purpose of detecting early pandemic outbreaks. Innovative advances in microfluidics, microelectron-mechanical systems technology, nanotechnology and 3D printing, as well as data analytics and development of efficient surface ligands have significantly facilitated the development of POCT diagnosis in the last two years. POCT is still in its infancy on a global scale, with technological advancements needing to be addressed in the future. This is also valid for an SPR-based sensor. While still mostly research-based instrumentations, portable surface plasmon resonance devices have proven to be of great value for the current SARS-CoV-2 pandemic. We hope to have shown here that some of the disadvantages of conventional SPR testing, such as bulky instrumentation and its difficult implementation in clinical settings, have been partially overcome with such miniaturized approaches. Their miniaturized nature combined with adequate surface architecture allow for their implementation in biosafety-level-3 conditions to screen novel bioreceptors for their affinity to different virus epitopes and results in a handful of sensitive SARS-CoV-2 diagnostic platforms. With reliable SPR tests down to 10 PFU/mL, they can be seen as alternative to lateral flow antigenic assays for which most reliable tests detect 50 PFU/mL equivalent to about 3 × 10^6^ RNA copies/mL. The possibility of multichannel and multianalyte analysis might offer SPR additional advantages in clinical settings. The clinical performance was tested more closely in at least one approach under an EU-funded project, CorDial-S. The evaluation of 119 nasopharyngeal swab samples achieved an 88% positive percentage agreement (PPA) and a 92% negative percentage agreement (NPA). The sensors could only be used one time, as the regeneration of the surface resulted in decreased performance, i.e., an 86% positive percentage agreement (PPA) and an 82% negative percentage agreement (NPA). Interestingly, the regeneration of the surface mainly had a large effect on the negative samples, with false positive responses obtained. Out of 50 negative samples screened on reused interfaces, 41 were assigned by RT-PCR and SPR as negative.

With these results at hand, what are the SPR perspectives in viral detection? The liquid sample volumes as well as power consumption of SPR-based biosensors remain the main bottlenecks for biomedical applications. To circumvent these drawbacks, improved and compact microfluid devices, as power-free pump systems, have to be considered for the next generation of integrated SPR-based biosensors. The use of sensing cartridges is one attempt taken by Affinité Instruments together with us to reduce the implementation of costly pumps. These disposable SPR sensors are low-cost and easy-to-use sensing devices intended for rapid single-point measurements. The integration of nanomaterials into SPR-based sensors needs to be pursued in this field if ultra-sensitivity becomes an important parameter. The integration of magnetic fields into SPR and the use of magnetic particles might be a way toward improved viral sensing. A magnetically enhanced SPR (M-SPR) was investigated lately (unpublished data) and showed to result in a detection limit as low as 1.5 × 10^3^ viral particles mL^−1^, two orders lower than the detection limit of conventional SPR, being 5.9 × 10^4^ viral particles mL^−1^. This and other concepts will allow driving the SPR field in the future.

It can be inferred that the plasmonic approach might also be adapted for the post-COVID crisis, notably for providing diagnostic parameters for distinguishing long-COVID patients from others. It is now recognized that many patients infected with SARS-CoV-2- can develop post-acute COVID syndromes a few months after the initial infection. This health stage, called long-COVID, occurs in 30–50% of COVID-19 patients and is characterized by multisystem symptoms, persistent fatigue and cogitative impairment more present with increasing age and female sex. In spite of the early impression that long COVID can only develop in patients who were hospitalized and intubated, increasing evidence indicates that long COVID can develop regardless of the severity of the original symptoms [70].
